# Dietary prevention of *Helicobacter pylori*-associated gastric cancer with kimchi

**DOI:** 10.18632/oncotarget.4897

**Published:** 2015-08-10

**Authors:** Migyeong Jeong, Jong-Min Park, Young-Min Han, Kun Young Park, Don Haeng Lee, Joon-Hwan Yoo, Joo Young Cho, Ki-Baik Hahm

**Affiliations:** ^1^ CHA Cancer Prevention Research Center, CHA Cancer Institute, CHA University, Seoul, Korea; ^2^ College of Nutrition, Busan National University, Busan, Korea; ^3^ Department of Gastroenterology, School of Medicine, Inha University, Incheon, Korea; ^4^ Digestive Disease Center, CHA University Bundang Medical Center, Seongnam, Korea

**Keywords:** cancer preventive kimchi, Helicobacter pylori, anti-inflammation, rejuvenation, anti-mutagenesis

## Abstract

To prove whether dietary intervention can prevent *Helicobacter pylori*-induced atrophic gastritis and gastric cancer, we developed cancer preventive kimchi (cpKimchi) through special recipe and administered to chronic *H. pylori*-initiated, high salt diet-promoted, gastric tumorigenesis mice model. *H. pylori*-infected C57BL/6 mice were administered with cpKimchi mixed in drinking water up to 36 weeks. Gross and pathological gastric lesions were evaluated after 24 and 36 weeks, respectively and explored underlying molecular changes to explain efficacies. Cancer preventive actions of anti-inflammation and anti-mutagenesis were compared between standard recipe kimchi (sKimchi) and special recipe cpKimchi in *in vitro H. pylori*-infected cell model. The erythematous and nodular changes, mucosal ulcerative and erosive lesions in the stomach were noted at 24^th^ weeks, but cpKimchi administration significantly ameliorated. After 36^th^ weeks, scattered nodular masses, some ulcers, and thin nodular gastric mucosa were noted in *H. pylori*-infected mice, whereas these gross lesions were significantly attenuated in cpKimchi group. On molecular analysis, significant expressions of COX-2 and IL-6, activated NF-κB and STAT3, increased apoptosis, and marked oxidative stresses were noted in *H. pylori*-infected group relevant to tumorigenesis, but these were all significantly attenuated in cpKimchi group. cpKimchi extracts imparted significant selective induction of apoptosis only in cancer cells, led to inhibition of *H. pylori*-induced proliferation, while no cytotoxicity through significant HO-1 induction in non-transformed gastric cells. In conclusion, daily dietary intake of cpKimchi can be an effective way either to rejuvenate *H. pylori*-atrophic gastritis or to prevent tumorigenesis supported with the concerted actions of anti-oxidative, anti-inflammatory, and anti-mutagenic mechanisms.

## INTRODUCTION

*Helicobacter pylori* (*H. pylori*) infection is considered as a major risk factor for gastric cancer, class I carcinogen, but it does not explain the whole picture of gastric carcinogenesis because additional modifications such as environmental or genetic factors, smoking, alcohol, diet, hygiene and bacterial or host genetic background are also implicated in carcinogenesis. There is no clear evidence to support cancer prevention through *H. pylori* eradication alone [1], although *H. pylori* infection is an important risk factor for gastric cancer. Dietary modification to inhibit carcinogenic pathways can be practical strategies for the prevention of gastric cancer in addition to eradication [2]. In gastric carcinogenesis, however, dietary factors themselves are dual-edged swords, implicated in carcinogenesis, but possibly preventive on others. For example, red and processed meat intakes were proven to be associated with an increased risk of gastric non-cardia cancer, whereas vegetable and fruits are protective factors especially in *H. pylori* antibody-positive subjects [3]. Although the cohort-based evidence is still lacking, a few dietary approach with antioxidants or nutraceuticals are available to prevent *H. pylori*-associated gastric diseases until now.

Kimchi is a traditional Korean fermented side-dish in which salt, spices, and other condiments have undergone lactic acid fermentation, in addition to high levels of vitamins, minerals, dietary fibers, and phytochemicals from the included ingredients [4]. A case-control study to assess the influence of Korean foods showed that kimchi significantly decreased the risk of gastric cancer, whereas intake of cooked rice with beans, charcoal grilled beef, pollack soup, and Dongchimi significantly increased the risks [5]. In a follow up study, they also found that gastric cancer risk could be decreased if those with *H. pylori* infection increase their intakes of antioxidant vitamins [6]. Just like yogurt and other fermented milk products had been important sources for probiotics in Western country, kimchi can be defined as “probiotic food” in Korea because it contains myriad types of probiotic lactobacillus, *L. plantarum* as a representative probiotic strain [7, 8]. Our previous study [9] showed that *L. plantarum* isolated from kimchi exerted significant anti-inflammatory actions against *H. pylori* infection. In addition to profuse probiotics, each ingredient included in kimchi recipe contained various components exerting anti-inflammatory, antioxidative, and anti-mutagenic actions like glucosinolate or glucoraphanin or isothiocyanate from cabbage, capsaicin from red pepper, isocyanate from radish and onion, gingerol from ginger, allicin from garlic, and β-carotene from carrot [10–13].

As a dietary intervention for *H. pylori* infection, we invented novel cancer preventive kimchi (cpKimchi) recipe and put hypothesis that dietary intervention of our cpKimchi can prevent *H. pylori*-associated gastric cancer in mice model. Generation of cpKimchi was based on the addition of mustard leaf, pear, mushroom, Chinese pepper, and sea tangle juice onto standardized kimchi recipe (see Supplementary table 1), which was chosen by our preliminary study.

## RESULTS

### Different biological actions of sKimchi and cpKimchi in *H. pylori*-infected cell model

**Figure 1:**
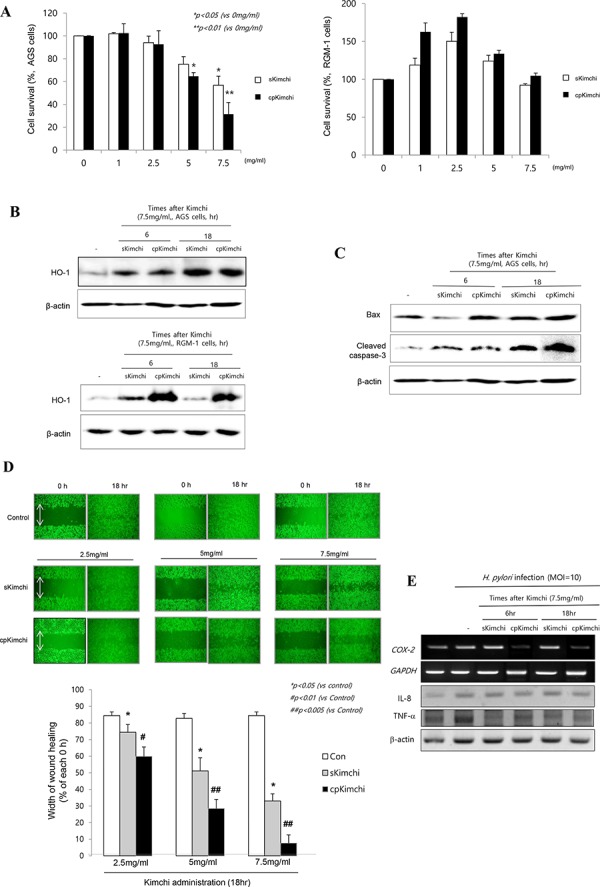
Biological actions of standard Kimchi (sKimchi) and cpKimchi; comparison in *in vitro H. pylori* cell model **A.** Cell survival by MTT assay MTT assay was done in AGS cells (left) and RGM-1 cells (right) under the challenge with 1, 2.5, 5, and 7.5 mg/ml concentration of sKimchi and cpKimchi soluble extracts, respectively. Significant cytotoxicities were noted with kimchi more than 5 mg/ml concentration only in AGS cells, none in RGM-1 cells even after kimchi more than 5 mg/ml **B.** Western blot of HO-1 after each kimchi extracts **C.** Western blot for Bax and cleaved caspase-3 **D.** Wound healing assay in AGS cells Previous study showed cpKimchi afforded selective cytotoxicity in cancer cells, wound healing assay after each kimchi extracts administration was done in AGS cells. Significantly delay in wound healing was noted in group administered with cpKimchi extracts. **E.** RT-PCR and Western blot for COX-2, IL-8, and TNF-α in the presence of *H. pylori* infection (MOI = 10). cpKimchi significantly attenuated *H. pylori*-induced COX-2 and TNF-α expression.

In two cell lines, AGS cells (Fig. 1A left) as gastric cancer cells and RGM-1 cells (Fig. 1A right) as non-transformed gastric epithelial cells, we checked the cell viability according to different dose of sKimchi and cpKimchi extracts, administered in four concentrations, 1, 2.5, 5, and 7.5 mg/ml, respectively. A dose of higher 5 mg/ml of kimchi extracts showed significant cytotoxicity in AGS cell (*p < 0.05*), whereas it did not show cytotoxicity in non-transformed gastric mucosal cell, RGM-1 at all (Fig. 1A). cpKimchi was superior in affording cytotoxicity compared to sKimchi (*p < 0.01*) in AGS cells. Similar results were also drawn from other gastric cancer cell lines, MKN28 and SNU-719 cells (see Supplementary Fig. 1A). Then, questions arose why cpKimchi induced selective cytotoxicity of transformed cancer, AGS cells, whereas no cytotoxicity was seen in non-transformed RGM-1 cells. In order to validate these superior and selective cytotoxicities of cpKimchi in cancer cell only, we checked the expression of heme oxygenase-1 (HO-1) after 6 and 18 hr, respectively. The expressions of HO-1 were significantly increased in both RGM-1 cells and AGS cells with cpKimchi, but the expressions of HO-1 were more prominent in RGM-1 cells (Fig. 1B). The expressions of HO-1 were also increased after sKimchi, but less compared to cpKimchi. We speculated the cytotoxicities of cpKimchi were blunted in non-cancer cells through the robust induction of cytoprotective gene, HO-1. We hypothesized that increased cytotoxicity in cancer cells, while no cytotoxicity in non-cancer cells of cpKimchi might be related with selective apoptotic action of cpKimchi, for which we checked the expression of Bax and cleaved capspase-3. As seen in Fig. 1C, cpKimchi showed increased expression of Bax and cleaved caspase-3 compared to sKimchi in AGS cells. As anticipated, cpKimchi didn't show any increased expression of Bax in RGM-1 cells (Supplementary Fig. 1B). All of these experimental results that cpKimchi selectively induced cytotoxicity in cancer cells, while no cytotoxicity in non-transformed cells were further validated with wound healing assay to find that cpKimch imposed capacity to limit the cell growth relevant to anti-tumorigenesis. As seen in Fig. 1D, the almost wound closure was achieved at control AGS cells after 18 hr, while 50% wound healing achieved with 5 mg/ml concentration of sKimchi and 35% with 7.5 mg/ml, suggesting that significantly lowered wound healing with sKimchi compared to control was noted at 18 hr (*p < 0.01*). However, more significant retarded wound healing was noted with cpKimchi extract treatment, only 30% wound closure with 5 mg/ml and only 10% wound closure with 7.5 mg/ml of cpKimchi (*p < 0.005*, Fig. 1D), signifying that cpKimchi drastically hindered normal wound healing. Interestingly, just like cytotoxicity assay, cpKimchi didn't show delayed wound healing in RGM-1 cells (Supplementary Fig. 1C). Together the finding that cpKimchi significantly decreased *H. pylori*-induced COX-2 and TNF-α as seen in Fig. 1E, we reached to the belief that long-standing administration of cpKimchi can afford significant rescuing actions from *H. pylori*-induced gastric damages including tumorigenesis.

### Ameliorating efficacy and mechanisms of cpKimchi in *H. pylori*-infected chronic atrophic gastritis

#### Attenuated chronic gastritis with 24 weeks dietary intake of cpKimchi

**Figure 2:**
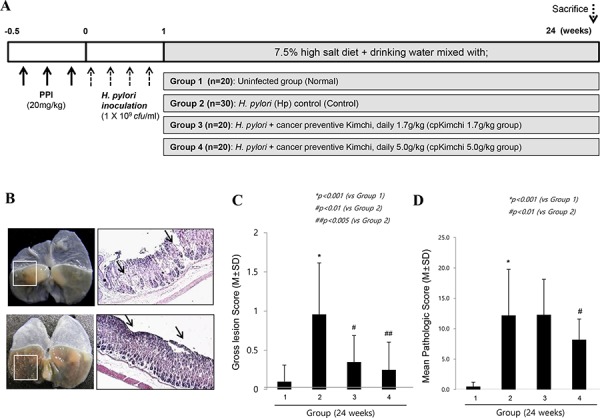
Ameliorating effects of cpKimchi in *H. pylori*-infected chronic atrophic gastritis (24 weeks after *H. pylori* infection) **A.** Protocol for *H. pylori*-associated gastritis model, 24 weeks. 90 mice were grouped into four, uninfected normal group (*n* = 20), *H. pylori*-infected control group (*n* = 30), 1.7 g/kg cpKimchi administered group (*n* = 20), and 5.0 g/kg cpKimchi administered group (*n* = 20) to document the efficacy of 24 weeks cpKimchi administration in *H. pylori*-initiated, high salt diet-promoted gastric damages model. **B.** Gross and pathological morphology and index according to group Administration of high salt diet after *H. pylori* infection led to accentuation of chronic atrophic gastritis. In detail, *H. pylori* infection followed with high salt diet led to some erosions, erythematous gastric mucosa, nodular mucosal changes, and protuberant foci of gastric mucosa at fore-stomach-glandular stomach area (box indicated). X100 **C.** Gross lesion scores according to group Gross lesion scores were significantly attenuated with cpKimchi administration (*p < 0.01*). **D.** Pathological scores according to group *H. pylori* infection for 24 weeks led to chronic atrophic gastritis presenting with loss of parietal cells, inflammatory cells such as monocytes, lymphocytes, and macrophages replacing gastric glands, and erosive mucosal changes. These changes were significantly decreased in group treated with cpKimchi 5 mg/kg (*p < 0.05*). The scoring system was described in “Materials and Methods”

After 24 weeks of *H. pylori* infection C57BL/6 mice (experiment scheme shown in Fig. 2A), co-administration of high salt diet after *H. pylori* infection led to accentuation of chronic atrophic gastritis and associated erosive gastritis as seen in Fig. 2B, on gross examination, *H. pylori* infection followed with high salt diet led to some erosions, erythematous gastric mucosa, nodular mucosal changes, and protuberant foci of gastric mucosa at forestomach-glandular stomach area. Gross lesion scores were significantly attenuated with cpKimchi administration (*p < 0.01*, Fig. 2C). As seen in Fig. 2B and 2D, the changes of chronic atrophic gastritis presenting with loss of parietal cells, inflammatory cells such as monocytes, lymphocytes, and macrophages replacing gastric glands, and erosive mucosal changes were prominent in 24 weeks of *H. pylori* infection. However, these changes were significantly decreased in group treated with cpKimchi 5 mg/kg (*p < 0.05*). We speculated that all of these beneficiary results were based on rejuvenating actions of cpKimchi.

#### Anti-inflammatory and rejuvenating actions of cpKimchi

**Figure 3:**
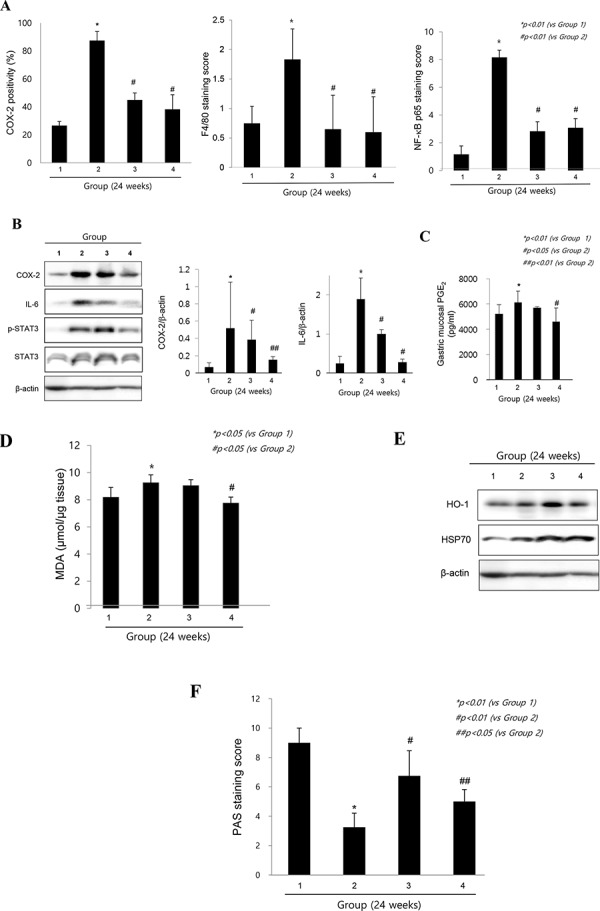
Molecular mechanisms explaining anti-inflammatory and rejuvenating action of cpKimchi (24 weeks after *H. pylori* infection) **A.** The changes of immunohistochemical stainings of COX-2, F4/80, and NF-κB p65 expression according to group The increased expressions of COX-2 were one of core pathogenic mechanisms after *H. pylori* infection. The expressions of COX-2 were significantly increased in control group (*p < 0.01*). Macrophage and monocytes were intensively increased with *H. pylori* infection. However, macrophage infiltrations were significantly decreased in group treated with cpKimchi (*p < 0.01*). When performed immunostaining with NF-κB p65, significantly increased expressions were noted in control group, whereas much attenuated expressions were seen in group treated with cpKimchi (*p < 0.01*). **B.** Changes of western blot of COX-2, IL-6 and STAT3 activation according to group On western blot analysis of mucosal COX-2 expression, COX-2 was significantly increased after *H. pylori* infection (*p < 0.01*), but its expressions were significantly decreased in group treated with cpKimchi (*p < 0.05*). In searching for other intervening factors, significantly increased IL-6 and STAT3 activation was seen in control group. cpKimchi significantly attenuated these levels of IL-6 and associated STAT3 activation (*p < 0.05*). **C.** PGE_2_ changes according to group Measuring PGE2 levels were done with ELISA and mucosal PGE_2_ levels were significantly decreased in group treated with cpKimchi 5 mg/kg (*p < 0.01*). **D.** MDA levels according to group Oxidative stress relevant to NF-κB activation was reflected with the levels of MDA, index of lipid peroxidation. *H. pylori* infection significantly increased the levels of MDA, but these levels were significantly decreased with cpKimchi administration (*p < 0.05*). **E.** Changes of HO-1 and HSP70 according to group As indirect index of oxidative stress, we have measured the expressions of HO-1 and HSP70, respectively and the expressions of HO-1 and HSP70 were significantly increased in group treated with cpKimchi (*p < 0.05*). **F.** Gastric mucins according to group The gastric mucin was significantly decreased after *H. pylori* infection (*p < 0.01*), but its levels were significantly maintained with cpKimchi administration.

The increased expressions of COX-2 had been acknowledged as one of core pathogenic mechanisms after *H. pylori* infection. As seen in Fig. 3A, the expressions of COX-2 were significantly increased in control group (*p < 0.01*). Macrophage and monocytes were intensively increased with *H. pylori* infection documented with immunostaining of F4/80 antibody, but their levels were significantly decreased in the group treated with cpKimchi (*p < 0.01*). Since all of these inflammatory mediators after macrophage infiltration were transcriptionally related with redox-sensitive transcriptional activation, we performed immunostaining with NF-κB p65 and found that significantly increased expressions of NF-kB in gastric mucosa of *H. pylori* infection were observed in control group, whereas much attenuated expressions were seen in group treated with cpKimchi (*p < 0.01*, Fig. 3A). On western blot analysis of mucosal COX-2 expression, COX-2 was significantly increased after *H. pylori* infection (*p < 0.01*), but its expressions were significantly decreased in group treated with cpKimchi (*p < 0.05*, Fig. 3B). In searching for other intervening factors, significantly increased IL-6 and STAT3 activation was seen in control group. cpKimchi significantly attenuated these levels of IL-6 and associated STAT3 activation (*p < 0.05*, Fig. 3B). Measuring PGE_2_ levels were done with ELISA and mucosal PGE_2_ levels were significantly decreased in group treated with cpKimchi 5 mg/kg (*p < 0.01*, Fig. 3C). Oxidative stress relevant to NF-κB activation was reflected with the levels of MDA, index of lipid peroxidation. *H. pylori* infection significantly increased the levels of MDA, but these levels were significantly decreased with cpKimchi administration (*p* < 0.05, Fig. 3D). As indirect index of oxidative stress, we have measured the expressions of HO-1 and HSP70, respectively and the expressions of HO-1 and HSP70 were significantly increased in group treated with cpKimchi (*p < 0.05*, Fig. 3E). Finally decrement of gastric neutral mucin might be either a result of atrophic gastric changes or chronic *H. pylori* infection. The gastric neutral mucins were significantly decreased after *H. pylori* infection (*p < 0.01*, Fig. 3F), but its levels were significantly maintained with cpKimchi administration.

### Long-term dietary intake of cpKimchi prevented *H. pylori*-induced gastric tumorigenesis

#### Cancer prevention with 36 weeks dietary intake of cpKimchi

**Figure 4:**
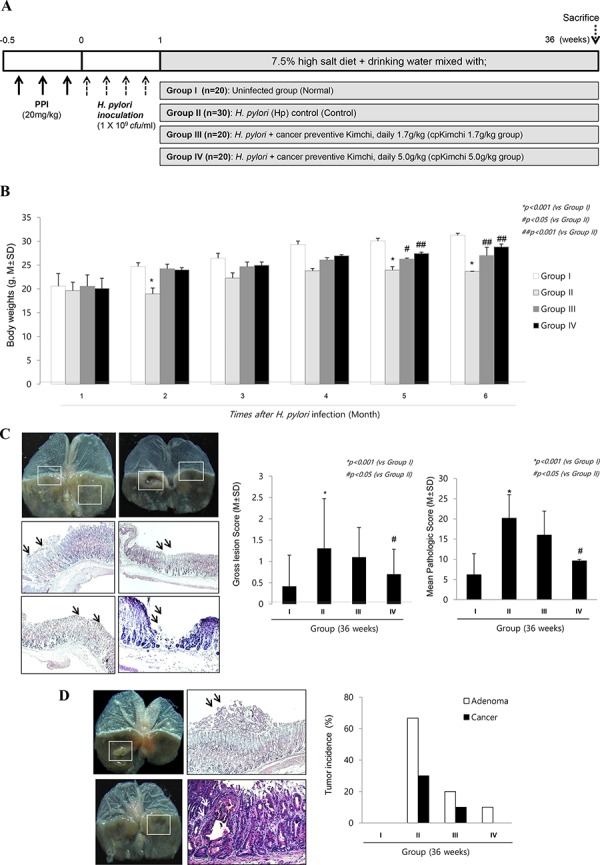
Prevention of *H. pylori*-induced gastric tumorigenesis with long-term intake of cpKimchi (36 weeks after *H. pylori* infection) **A.** Protocol for *H. pylori*-associated gastritis model, 36 weeks. In order to document the influence of cpKimchi onto the changes of tumorigenesis, the animal model was further extendedly observed up to 36 weeks **B.** Changes of body weights according to group Though the weight losses were significantly noted after *H. pylori* infection at 2 months post-infection (*p < 0.05*), significant weight changes were noted after 5–6 months, statistically significantly blocking of weight loss in group administered with 5 mg/kg cpKimchi (*p < 0.05*). **C.** Gross and pathological pictures and index according to group On gross evaluation of resected stomach, the following findings were obtained; nodular mucosal changes, thinned gastric mucosa, adenomatous polyps, and tumorous lesion with central ulcerations. The criteria for gross lesion score was shown in “Materials and Methods”. The whole gross lesion index was significantly increased in control group, but significantly decreased in group administered with cpKimchi (*p < 0.05*). Severe chronic atrophic gastritis, gastric ulcer, gastritis cystica profunda, adenoma, and adenocarcinoma were shown in control group. X 100. The mean pathological scores were significantly increased in control group, but significantly decreased in group administered with cpKimchi (*p < 0.05*). The scoring system was described in Supplementary table 2. **D.** The gastric cancer occurrence rate according to group Gastric adenoma and cancer occurrence were significantly increased in control group, but significantly decreased in group administered with cpKimchi.

In order to document the influence of cpKimchi onto the changes of tumorigenesis, the animal model was further observed up to 36 weeks (Fig. 4A). Though the significant weight losses were noted after *H. pylori* infection around 7 weeks, presumably related to *H. pylori*-associated gastric pathologies and increased leptin level, starting from 2 weeks and significantly after 29 weeks (*p < 0.001*), statistically significantly blocking of weight loss was noted in group administered with 5 mg/kg cpKimchi (*p < 0.05*, Fig. 4B). Our models, *H. pylori*-initiated and high salt diet-promoted, produced significant gastric tumors as well as severe degree of chronic atrophic gastritis at 36 weeks as seen in Fig. 4C and 4D, presented with findings including nodular mucosal changes, thinned gastric mucosa, adenomatous polyps, and tumorous lesion with central ulcerations. On pathological observation, these gross lesions were severe chronic atrophic gastritis, gastric ulcer, gastritis cystica profunda, adenoma, and adenocarcinoma. The whole gross lesion index was significantly increased in control group, but significantly decreased in group administered with cpKimchi (*p < 0.05*, Fig. 4C). Similar results noted in gross appearance were also drawn from pathological score analysis (*p < 0.01*, Fig. 4C). Our *H. pylori* infection model yielded significant tumorigenesis on gross observation in control group, which was proven to be gastric adenoma or gastric adenocarcinoma (Fig. 4D), but gastric tumorigenesis was significantly decreased with cpKimchi administration (*p < 0.01*, Fig. 4D). In Supplementary fig. 2, all representational pathological findings including gastric erosions, ulcer, chronic atrophic gastritis, some foci of intestinal metaplasia, dysplasia, and adenocarcinoma from Group II were presented.

#### Molecular mechanisms explaining cancer preventive effects of cpKimchi

**Figure 5:**
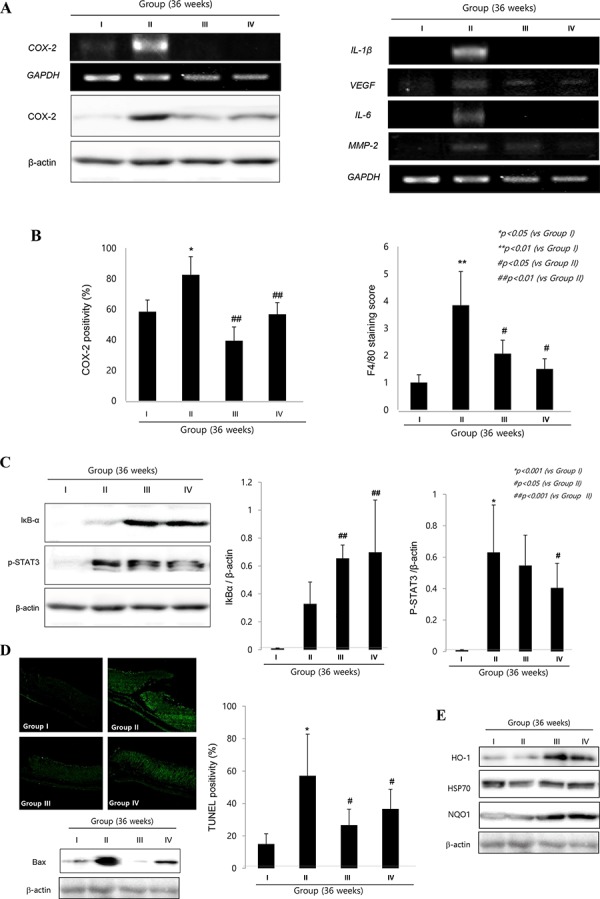
Molecular mechanisms to explain cancer preventive effects of cpKimchi (36 weeks after *H. pylori* infection) **A.** Changes of COX-2 and inflammatory mediators according to group On western blot and RT-PCR analysis of mucosal COX-2 expression, COX-2 was significantly increased after *H. pylori* infection, but its expressions were significantly decreased in group treated with cpKimchi. RT-PCR for IL-1β, VEGF, IL-6, MMP-2 was shown according to group and cpKimchi significantly decreased these *H. pylori*-induced inflammatory mediators. **B.** The immunohistochemical changes of COX-2 expressions and macrophage infiltrations according to group COX-2 and F/80 expressions were significantly increased in *H. pylori* infected control group. However, chronic 36 weeks intake of cpKimchi in drinking water significantly decreased COX-2 expressions as well as macrophage infiltration (*p < 0.01*). **C.** Western blot for p-STAT3 and IκB-α cpKimchi efficiently inhibited STAT3 activation and significantly inactivated IκB-α (*p < 0.001*). **D.** TUNEL staining for apoptosis and the expression of Bax In order to document the rejuvenating and restorative action of cpKimchi, TUNEL staining was done and apoptosis index was calculated according to group. Compatible with TUNEL, Bax expressions were significantly increased in Group II, but apparently decreased with cpKimchi administration. **E.** Western blot for Bax, NQO1, HO-1, and HSP70 according to group cpKimchi administration significantly increased NQO1 and HO-1 than control group II. HSP70 was significantly decreased in Group II, but preserved in Group III and IV.

Similar to the molecular changes observed at 24 weeks, COX-2 and F/80 expressions were significantly increased in *H. pylori* infected control group (Fig. 5A & 5B). Focused onto COX-2, as seen in Fig. 5A, *COX-2* mRNA as well as COX-2 expressions were significantly increased in Group II, but their levels were significantly decreased in Group III and Group IV (*p < 0.01*). Fig. 5B showed the mean changes of macrophage infiltrations according to group. Group II showed significantly increased infiltrations of macrophages (*p < 0.01*), but cpKimchi administration showed significantly decreased macrophage infiltrations (*p < 0.05*). On western blot and RT-PCR analysis of mucosal COX-2 expression, COX-2 was significantly increased after *H. pylori* infection, but its expressions were significantly decreased in group treated with cpKimchi (Fig. 5A). As a result, macrophage-related inflammatory mediators, including IL-1β, VEGF, IL-6, and MMP-2, all known to be engaged in either *H. pylori*-associated gastritis or carcinogenesis, were significantly increased in Group II. However, as noted in Fig. 5B, all of these macrophages-associated inflammatory mediators were significantly decreased in group II and IV, fortifying the suppressive actions of cpKimchi against *H. pylori*-associated macrophage infiltrations and activations. Especially, transcription factors engaged in these gastric inflammations, STAT3 and NF-κB, *H. pylori* infection significantly activated STAT3 and NF-κB activations (Fig. 5C). cpKimchi administration significantly inactivated NF-κB as reflected with increased levels of IκB-α (data not shown, but NF-κB p65 immunohistochemical stainings were significantly decreased in Group II and IV whereas significantly increased in Group II) as well as STAT3 phosphorylation. Since rejuvenating actions were imposed with cpKimchi that chronic atrophic gastritis was significantly decreased in Group 3 and 4 of 24 weeks observation (Fig. 2B), we have checked apoptosis and cytoprotective protein expressions according to group. Upon TUNEL staining to count apoptotic index, *H. pylori* infection was associated with significant bouts of apoptosis, but cpKimchi significantly attenuated apoptosis (Fig. 5D). Additional finding of Bax expression consistently showed increased expression at Group II. NQO1 and HO-1 are associated with antioxidative and cytoprotective function. Their expressions of NQO1 and HO-1 were significantly increased in Group III and IV (Fig. 5E).

## DISCUSSION

In the current study, we explored whether dietary intervention with kimchi intake can prevent *H. pylori*-associated gastric diseases including gastric cancer in mice model and found that long-term dietary administration of cpKimchi can be alternate way either to rejuvenate *H. pylori*-atrophic gastritis or to prevent *H. pylori*-associated gastric cancer. Detailed mechanisms of how cpKimchi prevented *H. pylori*-associated gastric cancer rendered high expectation of clinical achievement of dietary intervention to prevent troublesome *H. pylori*-associated gastric carcinogenesis, especially in high risk countries such as Korea. cpKimchi was made of additional supplementations of mustard leaf, Chinese pepper, pear, mushroom, and sea tangle juice instead of anchovy juice onto well-known kimchi recipe [14] through augmented significant rejuvenating actions or potentiated anti-mutagenic activities against *H. pylori* infection.

Gastric epithelial cells respond to *H. pylori* infection by up-regulating the expression of pro-inflammatory genes such as COX-2, iNOS, and IL-8, by which perpetuated gastric inflammation and overwhelmed oxidative stress led to significant DNA damage, robust apoptosis, and cell cycle dysregulation, all promoted gastric carcinogenesis [15, 16]. The additional fact that gastric inflammatory activities sustained even after complete removal of bacteria strongly supported the necessity of intervention of potent and sustained anti-inflammatory or antioxidative strategy for preventing *H. pylori*-associated gastric diseases [17, 18]. In general, high salt diet such as sodium chloride, consumption of nitrites, nitrates, alcohol, and pickled or smoked foods accelerate the development of stomach cancer, but natural products such as fresh vegetable and fruit or antioxidants such as beta-carotene, vitamin E, and vitamin C are known to suppress carcinogenesis [19–22]. However, core components of cpKimchi, some herbs and spices, have long been used by humans as foods and antidotes [12].

The common gradients of kimchi are pepper, garlic, black cumin, hyme, allspices, bay leaves, mustard leaf, rosemary containing carnosol, flaxseed in addition to Chinese cabbage, in which carotenoids, flavonoids, catechins, isothiocyanates, allicin, glucosinolate, and lignin are plentifully contained. We have added the following five ingredients (Supplementary table 1), pear, mushroom, red pepper, mustard leaf, and sea tangle juices upon common vegetables for kimchi in order to produce cpKimchi. One of core ingredients in cpKimchi, pear extracts, had been already reported to possess significant anti-mutagenic actions evidenced with anti-inflammation, anti-oxidation, and cell proliferation regulation. Feugang *et al* [20] have previously reported that aqueous extracts of pear significantly reduced gynecologic cancer cells growth through apoptosis induction and ROS-sensitive genes [21]. Though cranberry had the highest total phenolic content followed by apple, red grape, strawberry, pineapple, banana, peach, lemon, orange, pear, and grapefruit [22], these ingredients, except pear, are not usually included in kimchi. Next, special attention was paid to mushroom polysaccharides, since mushrooms are regarded as powerful pharmaceutical products against cancer. Numerous bioactive polysaccharides or polysaccharide-protein complexes from medicinal mushrooms had been described to either enhance innate and cell-mediated immune responses or exhibit antitumor activities in animals and humans [12, 23].

The third effective ingredient of cpKimchi was red pepper. Capsaicin, a pungent ingredient of red pepper, has been reported to possess antitumor activities as well as some direct anti-*H. pylori* action [10, 24]. The addition of red pepper powder into kimchi resulted in the slowing of the kimchi fermentation process during the early fermentation period and influenced the microbial succession and metabolite production during the kimchi fermentation processes [25]. The last two ingredients, mustard leaf and sea tangle juice also has been tried to promote cancer prevention. *Cleome viscosa Linn*. (Capparidaceae), commonly known as “wild mustard,” is an annual, sticky herb found as a common weed all over the plains of India and throughout the tropics of the world. The reported pharmacological actions of mustard leaf are anti-helmintic, anti-microbial, analgesic, anti-inflammatory, immunomodulatory, anti-pyretic, psychopharmacological, anti-diarrheal, and hepatoprotective activities [26]. Sea tangle juice has been reported to impose protective effect against ethanol and carbon tetrachloride-induced hepatic damage [27], lowering blood glucose in streptozotocin-induced diabetic rats [28]. Recently, Kang *et al* [23] performed a randomized, double-blind, and placebo-controlled clinical study to evaluate the antioxidant effects of fermented sea tangle on healthy volunteers with high levels of γ-glutamyltransferse and concluded that sea tangle enhanced the antioxidant defense system in a healthy population and may be useful as a functional food ingredient.

Excessive free radical generation overbalancing the rate of their removal leads to oxidative stress since oxidative stress has been principally implicated in the etiology of *H. pylori*-associated gastritis [29]. The antioxidative effects of red pepper/capsaicin, black pepper/piperine, ginger/gingerol, garlic/S-allyl cysteine, onion/anthocyanin & terpenoids, and cabbage/β-caretene relevant to cpKimchi all had been well-known [26, 30]. Furthermore, profuse lactobacillus, *L. plantarum*, nominated kimchi as probiotic food already. On the other hand, since kimchi is also preserved with salts, notoriety exists that kimchi might be also etiologic cuisine responsible for gastric cancer. However, fortunately, large scale cohort study performed in Korea [5] concluded that kimchi intake is beneficial in gastric cancer prevention. Since the salts concentration used in making cpKimchi was lowered a little than sKimchi from 2.5% to 2.2%, we speculated lowered salt did not affect gastric carcinogenesis. However, since we have adopted high salt (7.5%) diet model followed with *H. pylori* infection to promoted atrophic gastritis and subsequent gastric cancer, the influence of salt reduction was not consequential and did not influence overall outcome.

Even though there is still no clear answer whether *H. pylori* eradication can reverse the atrophy of the gastric mucosa and decrease the risk of gastric cancer development [31], a nation-wide intervention study to eradicate whole *H. pylori* in patients with chronic gastritis was already ongoing in Japan [8] to achieve cancer preventive effects of *H. pylori* eradication. In this background, we reached a conclusion that long-term dietary intervention with cpKimchi can be an alternate effort of high risk country like Korea to rescue from troublesome *H. pylori*-associated gastric carcinogenesis. However, well-designed clinical trials should be done to draw evidence based outcome of dietary intervention of cpKimchi administration for preventing *H. pylori*-associated gastric diseases including gastric cancer.

## MATERIALS AND METHODS

### Kimchi preparation

#### cpKimchi production

Preparation of kimchi was based on the standardized kimchi recipe of the Kimchi (sKimchi) Research Institute at Pusan National University [14]. As presented in Supplementary table 1, sKimchi is made of birned baechu cabbage (a kind of Chinese cabbage), red pepper powders, garlic, ginger, anchovy juice, sliced redish, green onion, some sugar, then fermented for some periods yielding lactobacillus like *L. plantarum*. In addition to these ingredients necessary for sKimchi production, additional supplements such as mustard leaf, Chinese pepper, pear, mushroom, and sea tangle juice instead of anchovy juice were included in cpKimchi (Supplementary Fig. 3); the taste and appearance were indiscernible between sKimchi and cpKimchi.

#### cpKimchi processing used for *in vitro* cell and *in vivo* animal model

All of the kimchi samples were freeze-dried and grounded into a fine powder. The kimchi powder underwent an extraction process with 20 times of methanol by stirring overnight. Finally, the kimchi methanol extracts were concentrated by heat evaporation (Büchi RE 111 rotavapor, Switzerland) and stored at 4°C as seen in Supplementary fig. 3. Since the usual serving dose of kimchi in Korea is approximately 30g-100g/day upon individual taste and about of 90% of general kimchi is composed of water, we could reduce the volume of kimchi through lyophilization and vaporization. The extracted cpKimchi was mixed into drinking water bottle, changed daily, in two serving dose for animal, 1.7 g/kg/day and 5.0 g/kg/day equivalent with usual general Korean intake dose of kimchi. Also cpKimchi and sKimchi extracts was dissolved into 2.5 mg/ml, 5 mg/ml, and 7.5 mg/ml in order to execute *in vitro* experiment.

### Bacteria culture

*H. pylori* strain ATCC 43504 (American Type Culture Collection, a *cag*A+ and *vac*A s1-m1 type's strain) was used for *in vitro* model and Sydney strain (SS1, a *cag*A+, *vac*A s2-m2 strain adapted for mice infection) for *in vivo* model. *H. pylori* were cultured at 37°C in BBL Trypticase soy (TS) agar plate with 5% sheep blood (TSAII; BD Biosciences, Franklin Lakes, NJ) under microaerophilic condition (BD GasPaK EZ Gas Generating Systems, BD Biosciences) for 3 days. The bacteria were harvested in clean TS broth, centrifuged at 3000 × g for 5 minutes, and resuspended in the broth at a final concentration of 10^9^ colony-forming units (CFUs)/mL. In all experiments, cultures grown for 72 h on TS agar plates were used.

### *H. pylori*-infected mice model

#### Animals

Five-week-old female C57BL/6 mice (Charles River Japan) were fed sterilized commercial pellet diets (Biogenomics, South Korea) and sterile water *ad libitum*, and housed in an air-conditioned biohazard room at a temperature of 24°C. After 1 week of adaptation, 20 mg/kg pantoprazole was injected three times to facilitate *H. pylori* colonization through lowered gastric acidity. And then each animal was intragastrically inoculated with a suspension of *H. pylori* containing 10^9^ CFUs/mL with an equal volume (0.1 mL) of clean TS broth using gastric intubation needles. All group were given injections of *H. pylori* total fourth times within a week. One group of 10 mice (uninfected group) was given injections of clean TS broth. The mice were fed a special pellet diet based on AIN76 containing 7.5% NaCl to generate more exacerbated data for 4 weeks. Then, *H. pylori* positive mice were randomly divided into four groups (*n* = 20). Pellet diet AIN76 containing 7.5% NaCl were administrated for 24 weeks and 36 weeks to promote *H. pylori*-induced carcinogenic process in all infected animals [32]. Experimental groups are shown in Fig. 2A and 5A, respectively and all animal studies were carried out in accordance with protocols approved by the Institutional Animal Care and Use Committee (IACUC) of CHA University CHA Cancer Institute after IRB approval. Stomachs were isolated and subjected to further histologic examination, ELISA, Western blotting, and RT-PCR.

#### Gross Index

After killing the animals, the isolated stomachs were cut open along the greater curvature and washed in ice-cold saline. To investigate the degree of gross mucosal damage, the mucosal sides of the stomachs were photographed using a digital camera and part of the mucosa was immediately fixed with 10% formalin solution. The gross damage of the gastric mucosa was assessed by three gastroenterologists, who were blinded to the treatments, using a gross ulcer index [33]. These lesions types were defined as follows; type I, presence of edema, hyperemia, or single submucosal punctiform hemorrhage; type II, presence of submucosal hemorrhagic lesions with small erosions; type III, presence of deep ulcer with erosions and invasive lesions.

#### Histopathology

For histopathological analysis, the stomach were fixed in 10% neutralized buffered formalin, processing using the standard method and embedded in paraffin. Sections of 4 μm thickness were then stained with hematoxylin and eosin (H&E) [34]. The glandular mucosae of corpus and antrum were examined histologically. The pathological changes of *H. pylori*-infection, such as inflammatory cells infiltration, erosive lesions, ulceration, dysplasia, adenoma formation (precancerous lesion), were graded by three gastroenterologists, who were blinded to the treatments, using an index of histologic injury defined [35]. In this study, inflammation was defined as grade the infiltration of inflammatory cells, 0: none, 1: under the lamina propria, 2: half of mucosa 3: until the epithelial gland layer (all mucosa). The erosion was defined as proportion of erosive lesion, 0: none, 1: loss of epithelial gland layer (1/3 proportion), 2: two-three portion of mucosa (2/3 proportion) 3: all mucosa (3/3 proportion) (Supplementary table 2)

#### Western blot analysis

Extracted stomach tissues were washed twice with PBS and then lysed in ice-cold cell lysis buffer (Cell Signaling Technology, Denver, MA) containing 1 mM phenylmethylsulfonyl fluoride (PMSF, Sigma Aldrich, St Louis, MO). After 20 min of incubation, samples were centrifuged at 10, 000 × *g* for 10 min. Supernatants were then collected. Proteins in lysates were separated by sodium dodecyl sulfate polyacrylamide gel electrophoresis (SDS-PAGE) and transferred to polyvinylidene fluoride (PVDF) membranes, which were incubated with primary antibodies, washed, incubated with peroxidase-conjugated secondary antibodies, rewashed, and then visualized using an enhanced chemiluminescence (ECL) system (GE Healthcare, Buckinghamshire, UK).

#### Immunohistochemical staining

After paraffin blocks were dewaxed and rehydrated with graded alcohol, tissue sections were heated in pressure jars filled with 10 mM citrate buffer in a microwave for 10 minutes. Slides were cooled in water for 15 minutes and washed in PBS. The slides were incubated overnight with the primary antibody. After incubation, a subsequent reaction was formed using a VECTOR kit (Vector Laboratories, Inc, Burlingame, CA). Finally, the slides were incubated with 3, 3ȃ-2-diaminobenzidine (Invitrogen Life Technologies) and counter-stained with hematoxylin (Sigma-Aldrich). Number of antibody positive cells was determined in 5 fields of the submesothelial area selected at random in each mouse and examined at × 100 magnification. Values are given as mean ± S.E.M.

#### PAS staining

For Periodic acid and Schiff's staining, histochemical staining of glycoconjugates was carried out as per the method of Pandurangan [36], using 2% periodic acid and Schiff (PAS)'s reagent in dark for 20 min. This resulted in a PAS staining score of between 10 (excellent preservation) and 0 (poor preservation).

#### TUNEL assay

To detect apoptosis, stomach tissues were stained with the terminal deoxynucleotidyl transferase-mediated dUTP nick-end labeling (TUNEL) method using the DeadEnd™ Fluorometric TUNEL System (G3250#, Promega, USA).

### *In vitro H. pylori*-infected cell model

#### Cell culture and cytotoxicity assay

Rat gastric epithelial cell lines (RGM-1) was given from Prof. H. Matsui (Tsukuba Univ., Japan) and AGS cells were purchased from ATCC (Manassas, VA), where the cells were properly stored and routinely authenticated (including DNA fingerprinting). After resuscitation in our lab, all the cells were used no longer than 6 months. AGS cells were cultured in RPMI-1640 medium (Gibco BRL, Gaithersburg, MD) and RGM-1 cells were cultured in DMEM medium. All mediums supplemented with 10% fetal bovine serum (Gibco BRL) at 37°C in 5% CO2. Cell viability was assessed using the MTT colorimetric assay. MTT [3-(4, 5-dimethylthiazol-2-yl)-2, 5-diphenyltetrazolium bromide] was purchased from Sigma Chemical Co. (St. Louis, MO). The filtrate (50 g) was mixed with water 1L and then lyophilized. The cells were plated into 96-well plates at 10^4^cells/mL and allowed to adhere for 24 h. Kimchi extract was applied in the test wells at various concentrations for 24 h.

#### Cell migration monitored with live cell image

AGS cells treated with dose-dependent sKimchi and cpKimchi were wounded with pipette tip and observed under ScopeTek MDC200 (CHA University, Seoul, Korea), in which cell growth was monitored up to 18 h and recorded with 3 min interval. Three different groups were monitored; No treat, dose-dependent treatment of sKimchi and treatment of cpKimchi. With the still photo taken after 18 h, the mean velocity of cell growth was calculated according to the group and the mean levels of cell migrations were displayed.

#### ELISA assay

Following harvesting of the stomach, and homogenized in 10 mM sodium phosphate buffer, pH 7.4 (1 mL). After centrifugation (9000 × *g*), the PGE_2_ level in the supernatant was measured by ELISA, and the concentration is expressed as pg/mg protein. The processes were performed according to Prostaglandin E_2_ express EIA kit manuscript (Cayman, Ann Arbor, MI).

#### Western blot for HO-1, Bax, PARP, and cleaved capspase-3

Extracted cells were washed twice with PBS and then lysed in ice-cold cell lysis buffer (Cell Signaling Technology, Denver, MA) containing 1 mM phenylmethylsulfonyl fluoride (PMSF, Sigma Aldrich, St Louis, MO). After 20 min of incubation, samples were centrifuged at 10, 000 × *g* for 10 min. Supernatants were then collected. Proteins in lysates were separated by sodium dodecyl sulfate polyacrylamide gel electrophoresis (SDS-PAGE) and transferred to polyvinylidene fluoride (PVDF) membranes, which were incubated with primary antibodies, washed, incubated with peroxidase-conjugated secondary antibodies, rewashed, and then visualized using an enhanced chemiluminescence (ECL) system (GE Healthcare, Buckinghamshire, UK).

### Statistical analysis

Results are expressed as the mean (standard deviation (SD). Statistical analyses were conducted with GraphPad Prism (GraphPad Software, La Jolla, CA, USA) and SPSS software (version 12.0; SPSS Inc., Chicago, IL, USA). Statistical significance between groups was determined by Mann-Whitney *U* test. Statistical significance was accepted at *p < 0.05*.

### Supplementary information

Detailed experimental procedures for reagents and RT-PCR can be found in Supplementary information Text.

## SUPPLEMENTARY MATERIALS & METHODS


