# Supplementary material for: Dietary prevention of Helicobacter pylori-associated gastric cancer with kimchi
Source: Oncotarget. 2015 Aug 10;6(30):29513–26. doi: 10.18632/oncotarget.4897 (PMC4745743; doi:10.18632/oncotarget.4897)
Supplement: Supplementary file 1 [file oncotarget-06-29513-s001.pdf]

SUPPLEMENTARY MATERIALS & METHODS

REAGENTS

All chemical reagents were obtained from Sigma (St. Louis, MO). MTT [3-(4,5-dimethylthiazol-2-yl)-2,5-diphenyltetrazolium bromide] was purchased from Sigma Chemical Co. (St. Louis, MO). Fetal Bovine Serum, penicillin/streptomycin, RPMI medium 1640 were obtained from Gibco BRL (Grand Island, NY). Western blotting detection reagents were obtained from Amersham Biotechnology (Bucks, UK). Primers for RT-PCR were synthesized by Bioneer (Daejeon, Korea). Reverse transcriptase was from Promega (Madison, WI). Antibodies for Bax, cleaved capspase-3, PARP, NF-κB p65, IκB-α, F4/80, IL-6, p-STAT3, STAT3, HSP70 antibodies were obtained from Cell signaling Technology Inc. (Beverly, MA), Inducible nitric oxide synthase (iNOS), cyclooxygenase-2 (COX-2), β-Actin, and vascular endothelial growth factor (VEGF) were from Santa Cruz Biotechnology (Santa Cruz, CA), Heme oxygenase-1 (HO-1) was from R&D Systems (Minneapolis, MN), and COX-2 was from Cayman Chemical (Ann Arbor, MI).

H. PYLORI-INFECTED MICE MODEL

RT-PCR

Total RNA was isolated from stomach tissues and cells using TRIzol reagent (Life Technologies, Milan, Italy), and 1–5 mg of each total RNA was transcribed to cDNA using the M-MLV Reverse Transcriptase (Promega, Madison, WI, USA) system for RT–PCR using Oligo-dt primer. Primers such as cyclooxygenase-2 (COX-2), vascular endothelial growth factor (VGEF), interleukin-1 beta (IL-1β), interleukin-6 (IL-6) and glyceraldehyde 3-phosphate dehydrogenase (GAPDH) were used for PCR. The PCR reactions were carried out for 35 cycles of 94°C for 30 s, 58°C for 30 s, 72°C for 30s, the primers for which are as follows: upstream 5’-CAT CCT GCC AGC TCC ACC GC-3’, downstream 5’-GGG AGG AAG GGC CCT GGT GT-3’for COX-2; upstream 5’-CCC TTC CTC ATC TTC CCT TC-3’, downstream 5’-GGG CAC CGA TCT GGG AGA GAG AG-3’ for VEGF; upstream 5’-CAG GCT CCG AGA TGA ACA ACA AAA -3’, downstream 5’-TGG GGA ACT CTG CAG ACT CAA ACT-3’ for IL-1β; and upstream 5’- CCG GAG AGG AGA CTT CAC AG -3’, downstream 5’- TGG TCT TGG TCC TTA GCC AC -3’for the IL-6; upstream 5’- AAT GTA TCC GTT GTG GAT CT -3’, downstream 5’- TCC ACC ACC CTG TTG CTG TA -3’ for GAPDH control.

MEASUREMENT OF STOMACH TISSUE LEVEL OF MDA

After sacrifice, collected tissue was used to measure the levels of MDA levels as a marker of oxidative stress (Oxis Research, Portland, OR, USA) according to manufacturer’s instruction. All samples were measured for their individual levels, and each sample was analyzed in triplicate manner, taking the mean of the three determinations.

IN VITRO H. PYLORI-INFECTED CELL MODEL

Cell culture

MKN28 and SNU-719 cells were given from Prof. S.J. Kim (CHA Univ., Korea), where the cells were properly stored and routinely authenticated (including DNA fingerprinting). After resuscitation in our lab, all the cells were used no longer than 6 months. MKN28 and SNU-719 cells were cultured in RPMI-1640 medium (Gibco BRL, Gaithersburg, MD). All mediums supplemented with 10% fetal bovine serum (Gibco BRL) at 37°C in 5% CO2.

Supplementary Table S1: Gradients of standard Kimchi and cancer preventive Kimchi.

Ingredients (g)	Standard Kimchi (sKimchi)	Cancer preventive Kimchi (cpKimchi)
Brined Baechu cabbage	100.0	100.0
Red pepper powder	3.5	2.5
Crushed garlic	1.4	2.8
Crushed ginger	0.6	0.6
Anchovy juice	2.2	–
Radish	13.0	11.0
Green onion	2.0	2.0
Sugar	1.0	1.0
Mustard leaf	–	7.5
Chinese pepper	–	0.1
Pear	–	2.8
Mushroom and sea tangle juice	–	5.0
Final salt concentration (%)	2.5	2.2

Supplementary Table S2: Determination of score in *H. pylori* - induced inflammation, ulcer, and atrophy.

	Scores
Inflammation	
1/3 portion of mucosa	1
2/3 portion of mucosa	2
3/3 portion of mucosa	3
Grade	
0 µm	0
0 ~ 1000 µm	1
1001 ~ 2000 µm	2
2001 ~ 3000 µm	3
3001 µm	4
Ulcers, Erosion length × depth (in mucosa)	
Gland	0.5
1/3 portion of mucosa	1
2/3 portion of mucosa	2
3/3 portion of mucosa	3
Ulcers size	
Ulecer or erosion up to 1 mm	N × 2
Ulcer of erosion larger than 1 mm	N × 3
Perforated ulcers	N × 4
Injury	
Discloration of mucosa	1
Edema	1
Hemorrhages	1
Number of petechia	
Until 10	2
More than 10	3
Atrophy	
<30% parietal loss	1
30–60% loss	2
>60% loss	3
Dyplasia	
Low-grade	N × 4
High-grade	N × 5
Adenoma	
Adenoma formation	N × 5

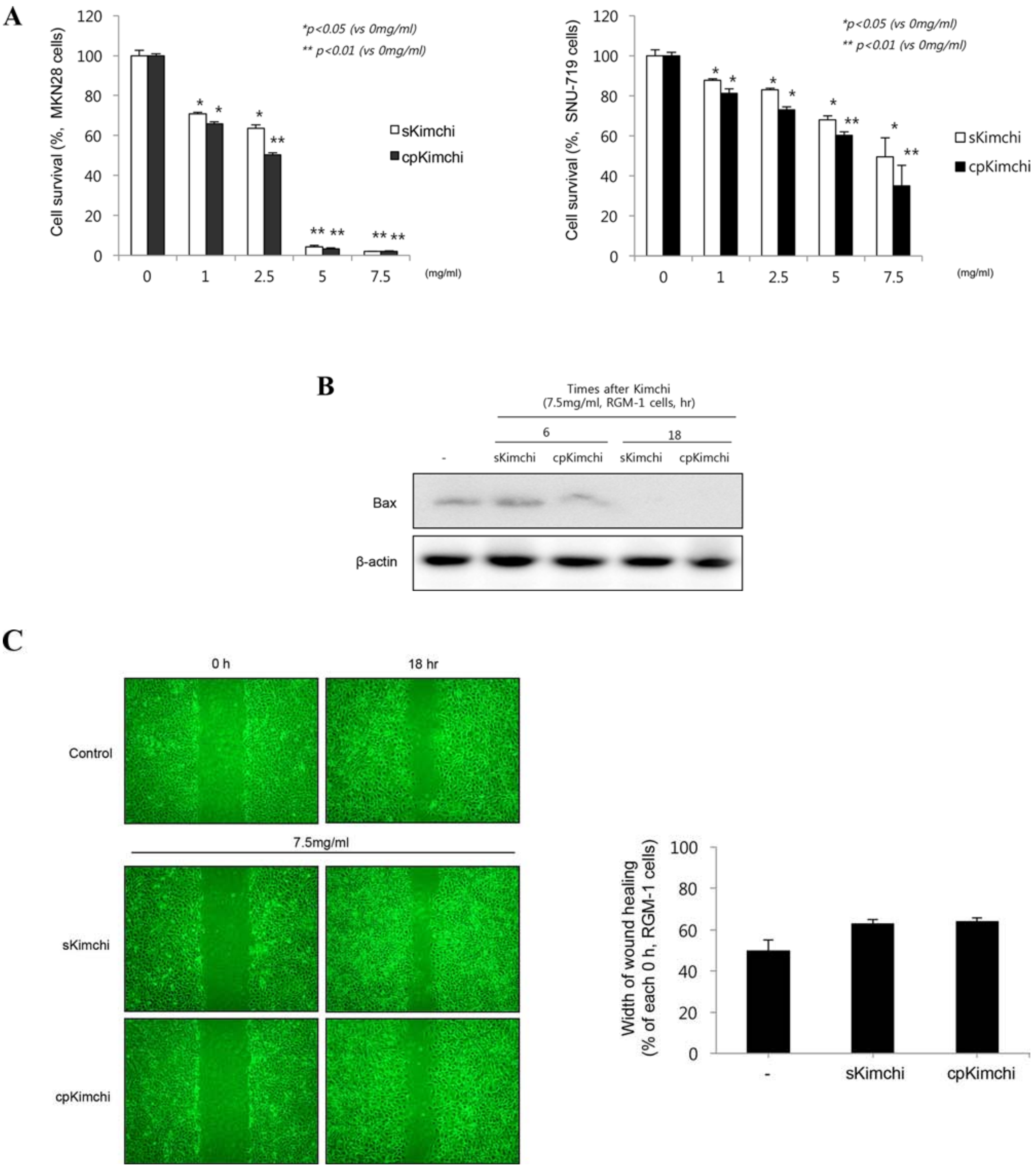

Supplementary Figure S1: Biological actions of standard Kimchi (sKimchi) and cpKimchi; comparison in *in vitro H. pylori* cell model. A. Cell survival by MTT assay MTT assay was done in MKN28 cells (left) and SNU-719 cells (right) under the challenge with 1, 2.5, 5, and 7.5 mg/ml concentration of sKimchi and cpKimchi soluble extracts, respectively. Significant cytotoxicities were noted with kimchi more than 1 mg/ml concentration in MKN28 cells and SNU-719 cells **B.** Western blot for Bax after each kimchi extracts **C.** Wound healing assay in RGM-1 cells Previous study showed Kimchi showed no cytotoxicity in RGM-1 cells, wound healing assay after each kimchi extracts administration was done in RGM-1 cells. No delay in wound healing was noted in group administered with Kimchi extracts.

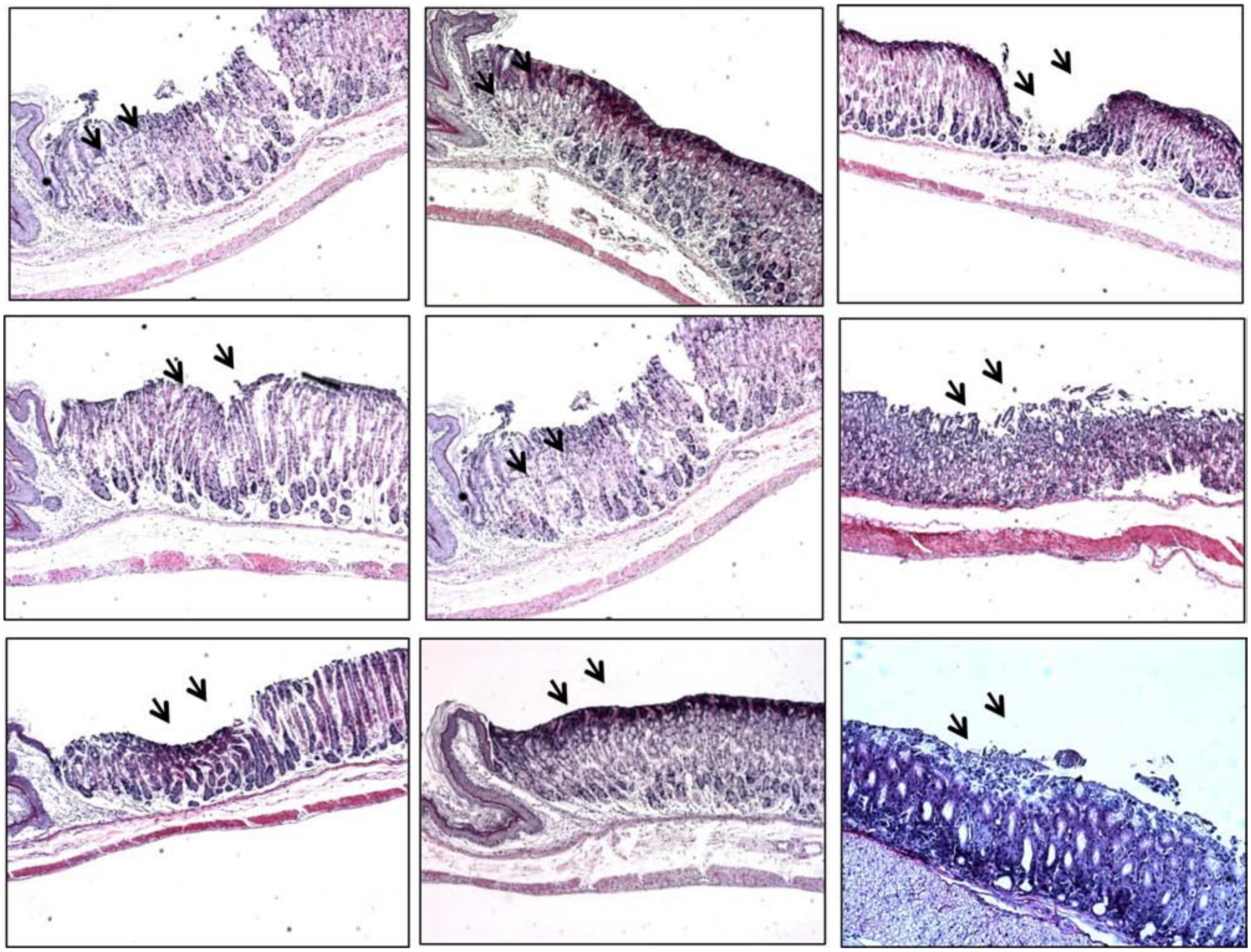

Supplementary Figure S2: Pathological findings of *H. pylori*-infected control group; gastric erosions, ulcer, atrophic gastritis, some foci of intestinal metaplasia, dysplasia, and adenocarcinoma were observed.

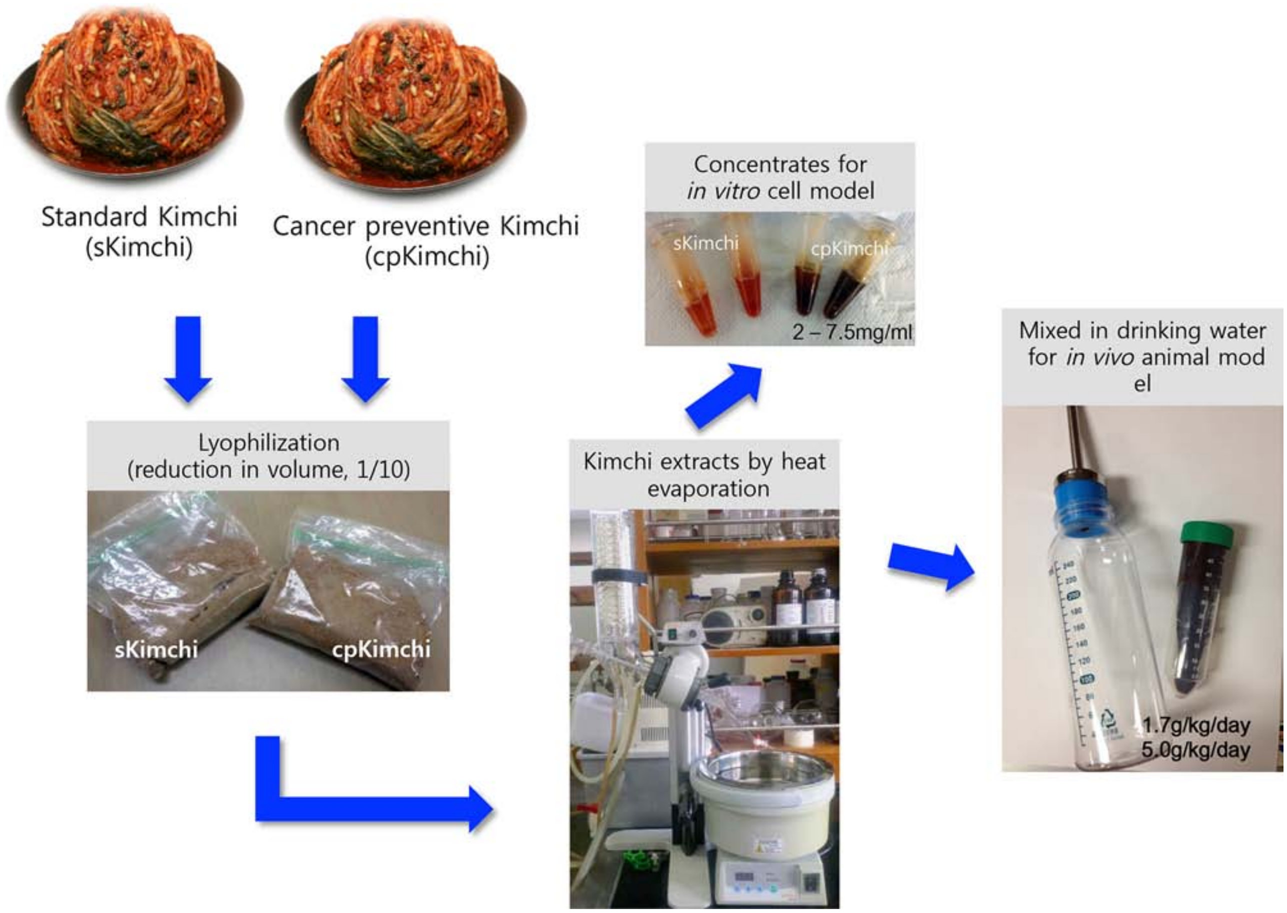

Supplementary Figure S3: cpKimchi processing for *in vitro* and *in vivo* experiment sKimchi and cpKimchi powder for final extracts. All of the kimchi samples were freeze-dried and ground into a fine powder. These were further concentrated by overnight stirring by heat evaporation (Büchi RE 111 rotavapor, Switzerland) to make 2 – 7.5 mg/ml for *in vitro* challenge and 1.7 g/kg and 5.0 g/kg Kimchi extracts for *in vivo* administration mized in drinking water They were stored at 4°C for experiment.
